# Effects of apelin and vascular endothelial growth factor on central retinal vein occlusion in monkey eyes intravitreally injected with bevacizumab: a preliminary study

**Published:** 2011-04-27

**Authors:** Tong Zhao, Qiang Lu, Yong Tao, Xiao-Ying Liang, Kai Wang, Yan-Rong Jiang

**Affiliations:** Department of Ophthalmology, People’s Hospital, Peking University, Key Laboratory of Vision Loss and Restoration, Ministry of Education, Beijing, China

## Abstract

**Purpose:**

To examine the intraocular distribution of bevacizumab at four weeks after intravitreal bevacizumab (IVB) injection and to investigate the effects of IVB on apelin and vascular endothelial growth factor (VEGF) in the central retinal vein occlusion (CRVO) of monkey eyes.

**Methods:**

Direct laser coagulation was performed on all branch retinal veins in the right eyes of six Rhesus monkeys to establish a CRVO model. The eyes of the first three monkeys were enucleated one week, two weeks, and 24 weeks after the establishment of the CRVO model; this was the CRVO group. Subsequently, IVB was injected into the eyes of the last three monkeys one week, two weeks, and 24 weeks after laser coagulation; this was the IVB group. The left eye of the first monkey was used as normal control. Immunohistochemistry and reverse-transcription PCR was used to examine the expression of apelin and VEGF. The penetration of bevacizumab into the retina and iris was investigated by fluorescence immunostaining.

**Results:**

Immunoreactivity for bevacizumab could be detected in the vessel walls of the iris and choroid on day 28 after injecting IVB: apelin and VEGF staining had been more prominent than normal in the CRVO eye, but these decreased following IVB injection. Expression of apelin mRNA (p<0.01) was lower in the IVB group than the CRVO group and did not vary significantly between groups.

**Conclusions:**

Bevacizumab could be detected in the iris and choroid after four weeks of intravitreal injection. Apelin may be partially suppressed by bevacizumab, and it may play a role in retinal neovascularization during the development of CRVO.

## Introduction

Central retinal vein occlusion (CRVO) is one of the most common retinal vascular diseases involving blindness [1]. Macular edema, caused by a decline in the blood–retina barrier, contributes to central vision loss. The decreased tissue perfusion leads to possible neovascular complications, such as rubeosis iridis and neovascular glaucoma, which can severely influence quality of life [2,3]. At present, photocoagulation has been widely used in CRVO to prevent neovascular complications. However, it cannot improve vision prognosis. Some evidence suggests that repeated intravitreal injections of triamcinolone may improve vision, but the complication of intraocular pressure and cataract makes it a less than ideal treatment [4].

The pathogenesis of CRVO is not very well understood and remains controversial. However, it is widely accepted that vascular endothelial growth factor (VEGF) plays an important role in CRVO development [5]. Anti-VEGF therapy, including intravitreal bevacizumab (IVB), has proven to be effective in improving visual acuity and inhibiting neovascularization [6,7]. However, research has revealed that anti-VEGF alone cannot completely prevent the occurrence of new vessels, which indicates that other factors may also participate in the process of neovascularization apart from VEGF [8]. Apelin is reported to act as an angiogenic factor that could stimulate the proliferation and migration of retinal endothelial cells and vascular tube formation [9,10]. That function cannot be replaced by VEGF [11]. Besides, recent studies suggest that apelin may be involved in retinal neovascularization during the development of proliferative diabetic retinopathy [12]. In an eye with CRVO, hypoperfusion causes stasis of the retinal bloodstream and retinal tissue hypoxia, which may induce upregulation of apelin, thereby simulating neovascularization.

To evaluate the potential effect of apelin in the pathogenesis of CRVO, we conducted the present study to examine whether bevacizumab could be detected four weeks after IVB and to investigate the expression of apelin in eyes with central retinal vein occlusion and the effect of bevacizumab.

## Methods

### Establishment of CRVO model

We established the CRVO model by obstructing all major retinal branch veins (usually two to three veins) of single eyes in six rhesus monkeys. All investigation involving animals conformed to the guidelines of the Association for Research in Vision and Ophthalmology's Resolution Statement for the Use of Animals in Ophthalmic and Vision Research. The veins were obstructed completely and permanently by mean of a green argon laser (Novus Omni system; Coherent Lambda Physik, Dieburg, Germany) with energy of 400–500 mW. Among the six eyes, five eyes received a second laser photocoagulation.

### Grouping of animals

Animals were numbered No. 1 to No. 6 and divided into different groups, as shown in Table 1. The left eye of No. 1 was used as the normal control. The right eyes of No. 1, 2, and 3 were enucleated one week, two weeks, and 24 weeks after the establishment of the CRVO model. These were used as the CRVO group and named “1 w,” “2 w,” and “24 w,” respectively, The right eyes of No. 4, 5, and 6, which were enucleated four weeks after intravitreal injection of bevacizumab, were defined as the IVB group and named “1 w+IVB,” “2 w+IVB,” and “24 w+IVB,” respectively. In the IVB group, No. 4, 5, and 6 differed in the intervals between the establishment of the CRVO model and intravitreous injection of bevacizumab, which were one week, two weeks, and 24 weeks, respectively.

**Table 1:** Grouping of animals

**Number**	**Eye**	**Time (w)**	**Group**	**Group name**	**Description**
1	OS	-	Control	Normal	-
1	OD	1*	CRVO	1 w	Photocoagulation
2	OD	2*		2 w	Photocoagulation
3	OD	24*		24 w	Photocoagulation
4	OD	4**	IVB	1 w + IVB	IVB at 1 week after photocoagulation
5	OD	4**		2 w + IVB	IVB at 2 weeks after photocoagulation
6	OD	4**		24 w + IVB	IVB at 24 weeks after photocoagulation

* represents the time (weeks) between creation of retinal vein occlusion and the enucleation. ** represents the time (weeks) between intravitreal injection of bevacizumab and the enucleation.

### Intravitreal injection of bevacizumab

Bevacizumab (Avastin; Roche, San Francisco, CA) with dosage of 0.05 ml (1.25 mg) was injected into the vitreous cavity under sterile conditions. Before injection, an anterior chamber puncture was made to maintain normal intraocular pressure. The animals were anesthetized with intramuscular injection of 30 mg/kg bodyweight ketamine hydrochloride (Ketalar, Parke-Davis, Morris Plains, NJ). Routine ocular examinations were done immediately after injection, in case of retina or lens injury. Levofloxacin Eye Drops (Santen Pharmaceutical Co.,Ltd., Ishikawa,Japan) were administered from this time point four times a day for 4 days to prevent infection. Animals were monitored for signs of inflammation until euthanasia with intramuscular injection of 10 mg/kg ketamine hydrochloride (Ketalar, Parke-Davis) followed by intravenous 100 mg/kg pentobarbital sodium (Sigma-Aldrich, St. Louis, MO).

### Fluorescence immunostaining

Eyes were enucleated, fixed in formalin, embedded in paraffin wax, sectioned, and deparaffinized. Goat fluoresceinisothiocyanate-conjugated anti-human immunoglobulin G (IgG; Zhongshan Goldenbridge Biotechnology, Beijing, China) was used to detect bevacizumab at a dilution of 1:50. Following incubation, the slides were washed and stained with 4’,6’-diamino-2-phenylindole (DAPI, No. D9542; Sigma-Aldrich, St. Louis, MO) at a dilution of 1:1000). The slides were examined with a fluorescence microscope (DS-Ril-U2; Nikon, Tokyo, Japan), and images were acquired with a digital camera (DS-U2, Nikon).

### Hematoxylin and eosin staining and immunohistochemistry

Routine hematoxylin and eosin (H&E) staining was performed. Immunohistochemistry was performed with rabbit anti-apelin polyclonal IgG (No. ab59469; Abcam, Cambridge, MA) at a dilution of 1:200 or mouse anti-VEGF polyclonal IgG (No. sc-7269; Santa Cruz Biotechnology, Santa Cruz, CA) at a dilution of 1:100. Biotin-conjugated goat antirabbit IgG, or biotin-conjugated goat antimouse IgG (Zhongshan Goldenbridge Biotechnology) were used as second antibodies, followed by DAB imaging. Photographs were acquired using a digital camera (DS-U2; Nikon) with the same camera settings (brightness, contrast, etc.)

### Detection of mRNA by reverse-transcription PCR

The embedded retinal tissue was sectioned, deparaffinized, and digested in proteinase-K (P6556; Sigma-Aldrich, St. Louis, MO). Total RNA was extracted from the retinal tissues of all groups. Two micrograms of RNA were converted into cDNA in a total reaction volume of 25 µl, and the reverse transcription product (1 µl) was then amplified by reverse-transcription PCR. The specific primers of human VEGF, human apelin and glyceraldehyde 3-phosphate dehydrogenase (GAPDH) were listed in Table 2. PCR products were electrophoretically separated on 2% agarose gel in a 1× tris-borate- EDTA (TBE) buffer. The optical density of each band was determined using BandScan software 4.5 (Glyko, Inc., Hayward, CA). For each band, five values were generated following the same procedure. The densitometric values for apelin and VEGF were normalized using *GAPDH* levels.

**Table 2:** The specific primers.

**Name**	**Primers (5′-3′)**	**Product length**
human apelin	F: CACCTCGCACCTGCTGTA	119 bp
	R: GAACGGGAATCATCCAAAC	
human vascular endothelial growth factor	F: TCCCCCTTGGGATCCCGCAG	91 bp
	R: GGCCGGGGAGGAGGTGGTAG	
Glyceraldehydes 3-phosphate dehydrogenase	F: GAGTCCACTGGCGTCTTCAC	120 bp
	R: GTTCACACCCATGACGAACA	

### Statistical analyses

The statistical analysis was performed using a commercially available statistical software package (Statistical Package for Social Sciences for Windows, version 17.0; SPSS, Chicago, IL). Tests for independent samples were performed to compare differences in densitometric values for apelin or VEGF. Two-tailed probabilities of less than 0.05 were considered to indicate statistical significance.

## Results

### Establishment of the central retinal vein occlusion model

**Figure 1:**
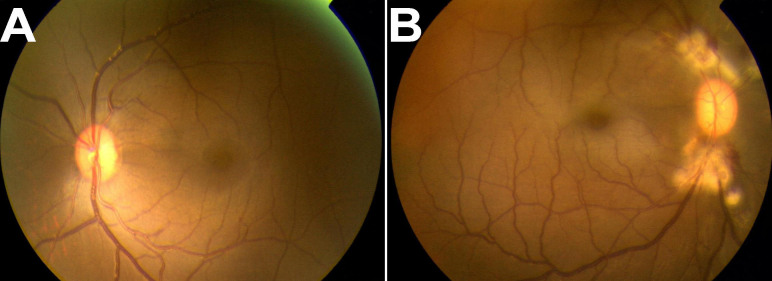
Photographs of fundus. **A**: This shows the normal fundus. **B**: Central retinal vein occlusion (CRVO) model was successfully established by photocoagulation. The fundus photograph shows photocoagulation spots and dilated retinal veins at the first day after photocoagulation.

The CRVO model was successfully established. On the first day after photocoagulation, fundus photograph showed laser photocoagulation spots and dilated retinal veins (Figure 1B), compared to normal fundus (Figure 1A).

**Figure 2:**
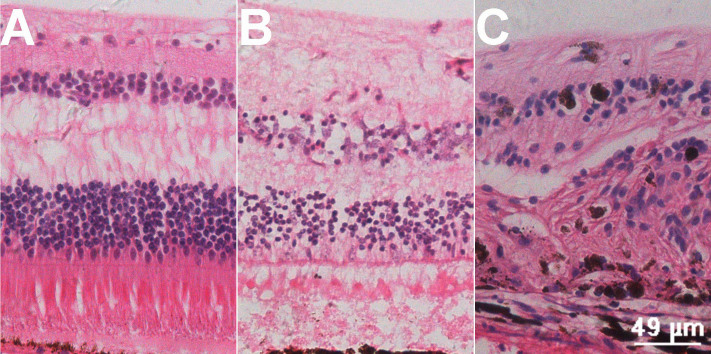
Hematoxylin and eosin staining section. With the establishment of central retinal vein occlusion (CRVO) model, the pathological changes of retina were remarkable. **A**: This image shows the normal retina structure. **B**: This image shows interstitial edema of the retina 7 days after photocoagulation. **C**: This image shows disordered retinal structure 24 weeks after photocoagulation.

A normal H&E section is shown in Figure 2A. Acute retinal edema was remarkable at 7 days after photocoagulation (Figure 2B). Disordered retinal structure was observed 24 weeks after the establishment of the CRVO model (Figure 2C).

### Fluorescence immunostaining of bevacizumab

**Figure 3:**
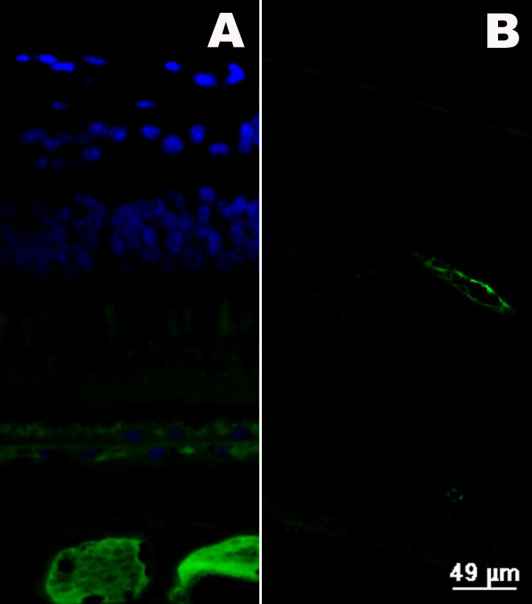
Fluorescence immunostaining of eye tissues after inravitreal bevacizumab (IVB) injection. 4’,6’-diamino-2-phenylindole (blue) was used to show nuclei and positive staining of bevacizumab (green) was showed in blood vessels. **A**: Bevacizumab (green) was detected in choroid vessels four weeks after IVB. **B**: Bevacizumab (green) was detected in the walls of iris vessel four weeks after IVB.

In the IVB group, bevacizumab was detected in the iris and choroid vessels (Figure 3) of all eyes at four weeks after intravitreous injection. There was no obvious difference among the three groups of “1 w+IVB,” “2 w+IVB” and “24 w+IVB.”

### Immunohistochemistry of vascular endothelial growth factor

**Figure 4:**
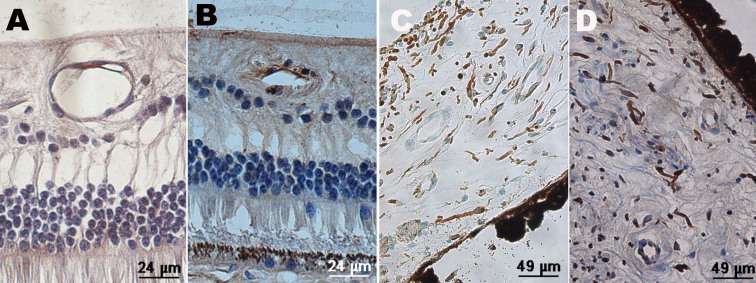
Immunochemistry of a normal monkey eye. Sections from normal retina and iris were examined by immunohistochemistry with anti-vascular endothelial growth factor (VEGF) antibody and anti-apelin antibody. **A**: Positive staining (brown) of VEGF was showed in the retinal vessel walls. **B**: Positive staining (brown) of apelin was showed in the retinal vessel walls. **C**: There was no positive staining of VEGF in the section of iris. **D**: There was no positive staining of apelin in the section of iris.

In normal monkey eyes, VEGF was detected in retinal vessel walls (Figure 4). In the iris tissue, there was no positive finding.

**Figure 5:**
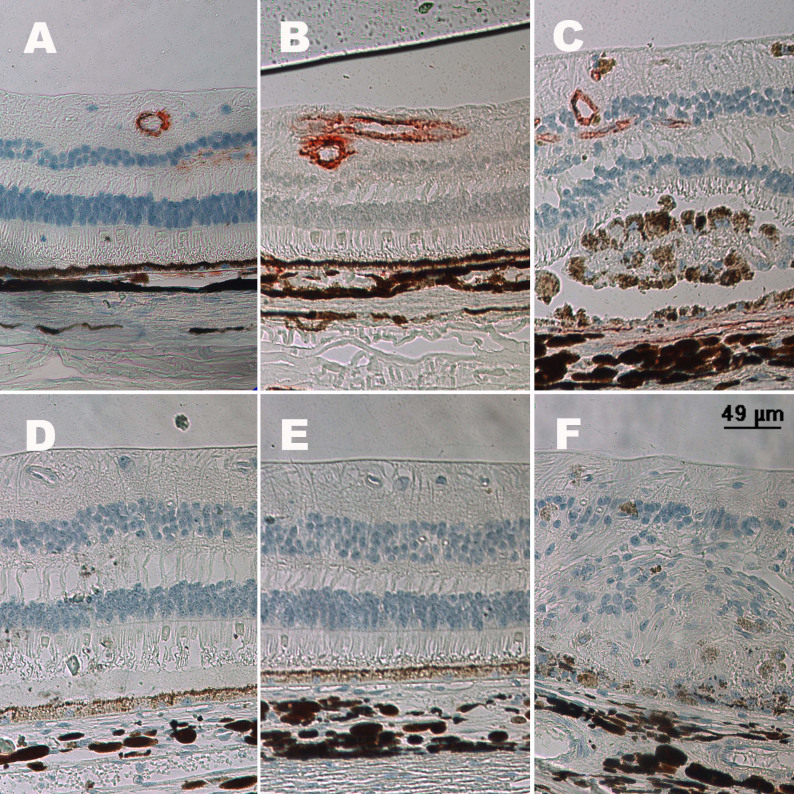
Immunochemistry of vascular endothelial growth factor in a retina. Sections of retina were examined by immunohistochemistry with anti-vascular endothelial growth factor (VEGF) antibody. Positive staining (brown) of VEGF was detected in retinal vessel walls in the central retinal vein occlusion groups (**A**: group “1 w,” **B**: group “2 w,” **C**: group “24 w”). There was no obvious positive staining after intravitreal bevacizumab (IVB) injection (**D**: group “1 w+IVB,” **E**: group “2 w+IVB,” **F**: group “24 w+IVB”).

**Figure 6:**
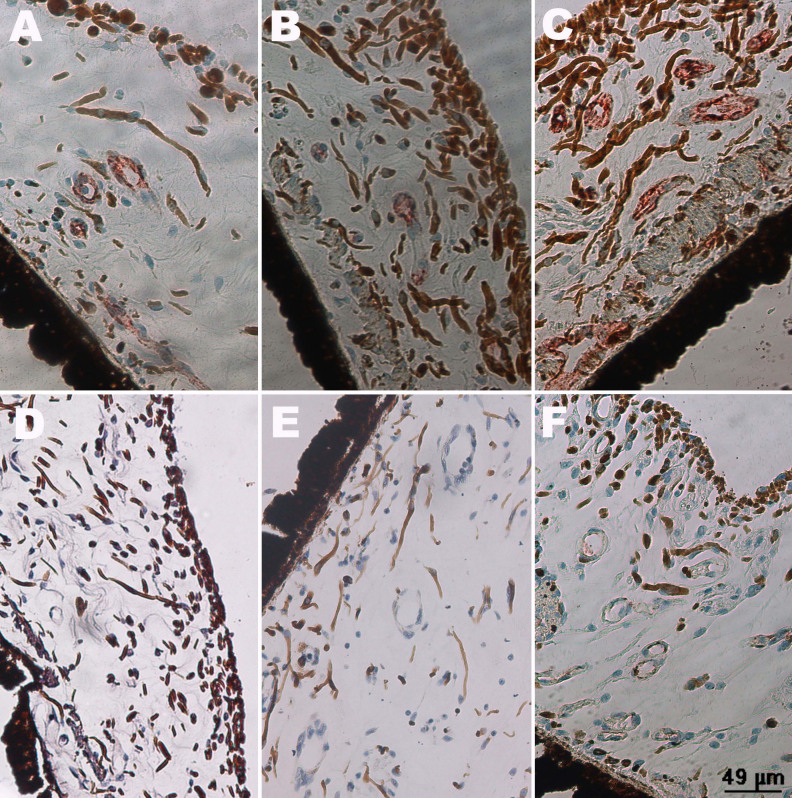
Immunochemistry of vascular endothelial growth factor in an iris. Sections of iris were examined by immunohistochemistry with anti-vascular endothelial growth factor (VEGF) antibody. Positive staining (brown) of VEGF was detected in vessel walls of iris in the central retinal vein occlusion groups (**A**: group “1 w,” **B**: group “2 w,” **C**: group “24 w”). There was no obvious positive staining after intravitreal bevacizumab (IVB) injection (**D**: group “1 w+IVB,” **E**: group “2 w+IVB,” **F**: group “24 w+IVB”).

As to CRVO groups, VEGF was detected in retinal vessel walls (Figure 5A-C). By contrast, there was no obvious positive staining in the IVB groups. In group “24 w+IVB,” in addition to VEGF-positive staining, disordered retinal structure and increased vessels were found. In the iris, VEGF was also detected in CRVO groups, and the amount of vessels increased with the prolongation of the course of the disease (Figure 6A-C). In the IVB group, VEGF could not be detected (Figure 6D-E).

### Immunohistochemistry of apelin

**Figure 7:**
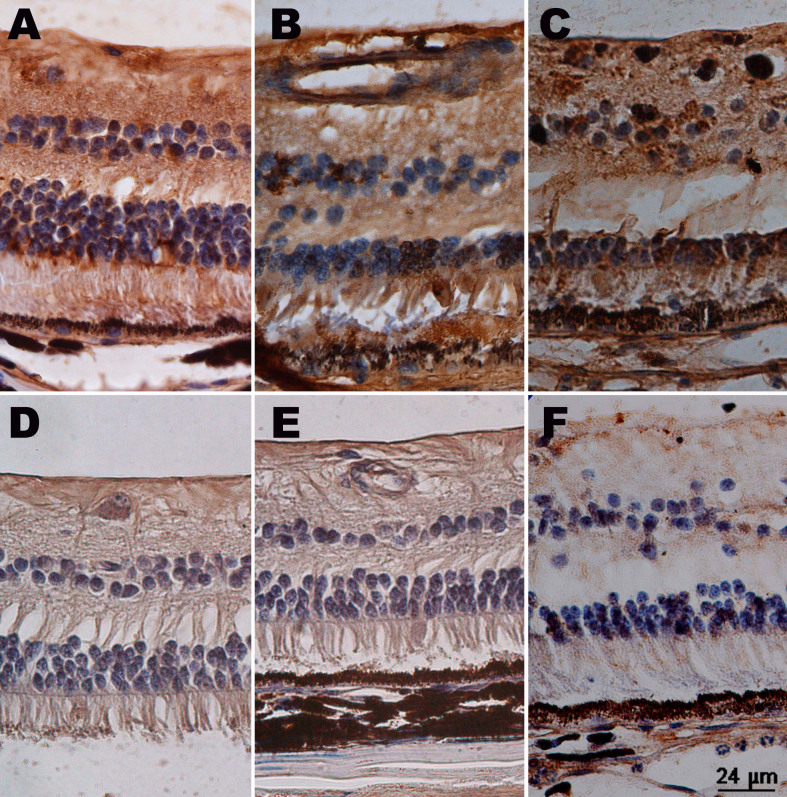
Immunochemistry of apelin in a retina. Sections of retina were examined by immunohistochemistry with anti-apelin antibody. Positive staining (brown) of apelin was detected in the retinal vessel walls, inner nuclear layer, and outer nuclear layer in the central retinal vein occlusion groups (**A**: group “1 w,” **B**: group “2 w,” **C**: group “24 w”). Among the intravitreal bevacizumab (IVB) groups (**D**: group “1 w+IVB,” **E**: group “2 w+IVB,” **F**: group “24 w+IVB”), only “24 w+IVB” group had positive staining of apelin in nuclear layers.

**Figure 8:**
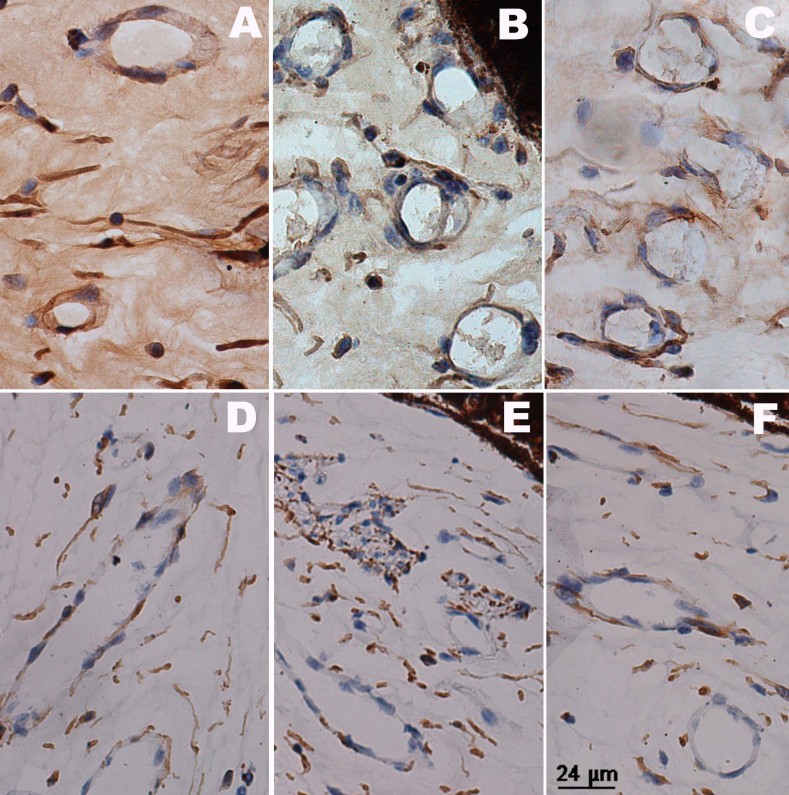
Immunochemistry of apelin in an iris. Sections of iris were examined by immunohistochemistry with anti-apelin antibody. Positive staining (brown) of apelin was detected in iris vessel walls in the central retinal vein occlusion groups (**A**: group “1 w,” **B**: group “2 w,” **C**: group “24 w”), and there was no obvious positive staining in the intravitreal bevacizumab (IVB) groups (**D**: group “1 w+IVB,” **E**: group “2 w+IVB,” **F**: group “24 w+IVB”).

Similar to VEGF, apelin was detected in normal retinal vessel walls but not in the iris (Figure 4). Apelin-positive staining was seen in the retinal inner nuclear layer, outer nuclear layer, and ganglion cells (Figure 7A-C), apart from the vessel walls. The prolongation of the course of the disease seemed to have nothing to do with apelin staining. In the IVB groups, decreased apelin staining was observed, and the distribution was similar to that of the normal eyes, except for the “24w+IVB” group (Figure 7D-E). Apelin was stained in the iris blood vessels, and expression was down-regulated after IVB injection (Figure 8).

### Expression of mRNA of vascular endothelial growth factor and apelin

**Figure 9:**
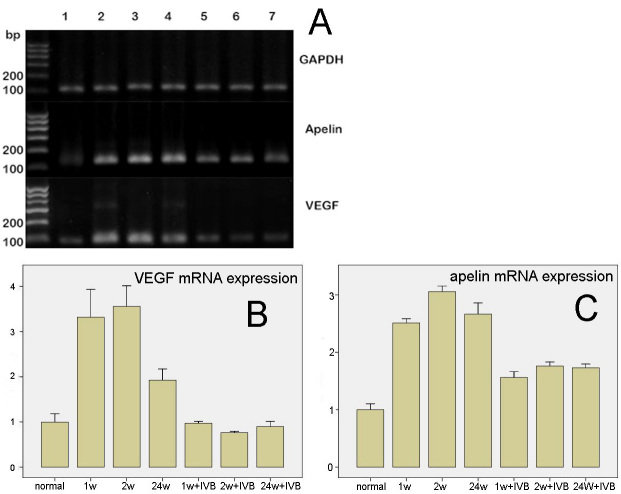
The expression of apelin and vascular endothelial growth factor mRNA. **A:** This shows the result of reverse-transcription PCR of *GAPDH*, apelin and vascular endothelial growth factor (VEGF). 1–7 represent the following groups: “normal,” “1 w,” “2 w,” “24 w,” “1 w+intravitreal bevacizumab (IVB),” “2 w+IVB,” and “24 w+IVB,” respectively. **B:** This shows the VEGF mRNA expression levels in the control, central retinal vein occlusion (CRVO), and IVB groups. Error bars represent SD. In all stages for the CRVO groups, the expression of mRNA of VEGF was upregulated, compared to the normal group (p<0.01). In the “24 w” group, the expression of VEGF mRNA was lower than in the “1 w” and “2 w” groups, but still higher than normal (p<0.01). After IVB, VEGF mRNA of all the three CRVO groups decreased significantly (all p<0.01); however, the VEGF mRNA level did not vary significantly between “1w+IVB” group, “2w+IVB” group and “24w+IVB” group and normal control (p=0.71, 0.12, and 0.24, respectively). **C:** Apelin mRNA expression levels in the control, CRVO, and IVB groups. Error bars represent SD. The expression of apelin mRNA in the CRVO groups was significantly higher than normal (p<0.01). After IVB, apelin mRNA of all the three CRVO groups decreased significantly (all p<0.01); the apelin mRNA level of “1w+IVB” group, “2w+IVB” group and “24w+IVB” were still significantly higher than normal control (all p<0.01).

The results of the PCR from the retinal tissue of each group are shown in Figure 9A. Expression of VEGF and apelin mRNA was observed in the normal monkey eye. In all stages for the CRVO groups, the expression of VEGF mRNA was upregulated, compared to the normal group (p<0.01). In the “24 w” group, the expression of VEGF mRNA was lower than in the “1 w” and “2 w” groups, but still higher than normal (p<0.01). After IVB, VEGF mRNA of all the three CRVO groups decreased significantly (all p<0.01); however, the VEGF mRNA level did not vary significantly between “1w+IVB” group, “2w+IVB” group and “24w+IVB” group and normal control (p=0.71, 0.12, and 0.24, respectively; Figure 9B). The expression of apelin mRNA in the CRVO group was significantly higher than in the normal eyes (p<0.01). In the IVB group, the expression of apelin mRNA was lower than in the CRVO group (p<0.01), but was still higher than normal (p<0.01; Figure 9C).

## Discussion

Previous studies have indicated that bevacizumab can penetrate quickly into all layers of the retina and iris after intravitreal injection [13,14]. Some researchers revealed that bevacizumab could be detected two weeks after intravitreal injection [15,16]. In this study, it was found that bevacizumab was stained in choroid vessels and iris vessel walls four weeks after intravitreal injection. This result confirmed the accumulation location of bevacizumab, consistent with previous studies, and prolonged the recognized time of bevacizumab immunoactivity after intravitreal injection. We speculate that gelatinous vitreous functions as a kind of sustained release system for the diffusion of bevacizumab. On the other hand, disturbed circulation in local blood vessels and slow perfusion may also contribute to the accumulation of bevacizumab in some vessels [14]. Further research about the distribution of bevacizumab in different diseases will be helpful in determining the optimum interval between repeated intravitreal injections of bevacizumab.

The results of immunochemistry indicated that VEGF was down-regulated after intravitreal injection of bevacizumab, which agreed with previous reports that intravitreal bevacizumab lowered the concentration of VEGF in aqueous fluid and vitreous [17-19]. However, research findings have varied on the effects of bevacizumab on *VEGF* mRNA expression [20,21]. We found that the expression of *VEGF* mRNA decreased four weeks after intravitreal injection of bevacizumab. These results suggest bevacizumab improves the ischemic state and subsequently lowers the secretion-inducing pressure of VEGF. The upregulation of *VEGF* mRNA immediately after intravitreal injection is supposedly related to feedback regulation, due to the sharp drop in VEGF concentration from bonding with bevacizumab.

Apelin was first extracted in 1998 [9]. It has been reported to stimulate the proliferation and migration of retinal endothelial cells, as well as to promote vascular tube formation [10]. Research implies that apelin might also participate in regulating new blood vessel growth in embryonic angiogenesis and tumor growth [11,22-24]. A study on the role of apelin in diabetic retinopathy showed there was a high vitreous concentration of apelin in eyes with proliferative diabetic retinopathy, as shown by immunofluorescence staining of apelin in the endothelial cells of the fibrovascular membranes of patients with proliferative diabetic retinopathy [12]. The above findings indicate that apelin might be an angiogenic factor that plays an important role in the pathogenesis of vascular disease, including retinal vein occlusion.

Our results also showed that apelin was expressed in the vascular system, which was consistent with previous studies [12]. It was further found that in the CRVO eyes, apelin was also stained in the inner and outer nuclear layers. It has been speculated that the cells of the inner and outer nuclear layers, apart from vascular endothelial cells stimulated by hypoxia, secrete apelin in the manner of autocrine and paracrine [11]. Apelin sequentially affected the endothelial cells, promoting angiogenesis and proliferation. Apelin may play an important role in the development of CRVO. We observed that after intravitreal injection of bevacizumab, 1) apelin staining decreased, 2) the expression of apelin mRNA was down-regulated, and 3) the down-regulation did not return to normal levels, indicating that apelin expression may be suppressed by anti-VEGF therapy; but the extent of suppression is not parallel with that of VEGF. Apelin may be partially regulated with VEGF, to some extent. At the same time, apelin has an independent, upstream signaling pathway. It was also observed that apelin was still detected in the inner and outer nuclear layers in the “24 w” group, instead of in the “1 w” or “2 w” groups. We speculate that in the earlier stage, the effect of hypoxia on the cells of the inner and outer nuclear layers is reversible, and can be eliminated when anti-VEGF therapy improves hypoxia. In late-stage CRVO, cells of the inner and outer nuclear layers are irreversibly injured, and begin releasing apelin persistently.

There remain some potential limitations in our study. First, this is a preliminary study. Due to the small number of animals and lack of fresh tissue, some tests were not performed, such as western blotting, which would have provided more-accurate quantitative evidence. Second, rhesus monkeys were used as the experimental model. We cannot exclude the possibility that the humanized antibody bevacizumab interfered with the monkey’s immunology. More evidence needs to be produced to translate these conclusions from monkey to humans. Third, the investigation of the interaction between apelin and VEGF did not involve the molecular signaling level. Our deductions lack sufficient experimental support. In addition, previous studies revealed that apelin mainly promoted the growth of immature vessels. A study on the earlier acute stage of CRVO may be necessary and important.

In conclusion, bevacizumab could be detected after four weeks of intravitreal injection. Apelin staining was prominent, and the expression of apelin mRNA was significantly higher in the CRVO group. Intravitreal injection of bevacizumab down-regulated the expression of apelin mRNA, to some extent. These results suggest that apelin may play a role in the development of CRVO, and that apelin has a unique upstream signaling pathway, independent of the VEGF pathway.
